# EST79232 and EST79376, Two Novel Sigma-1 Receptor Ligands, Exert Neuroprotection on Models of Motoneuron Degeneration

**DOI:** 10.3390/ijms23126737

**Published:** 2022-06-16

**Authors:** Núria Gaja-Capdevila, Neus Hernández, Sandra Yeste, Raquel F. Reinoso, Javier Burgueño, Ana Montero, Manuel Merlos, José M. Vela, Mireia Herrando-Grabulosa, Xavier Navarro

**Affiliations:** 1 Department of Cell Biology, Physiology and Immunology, Institute of Neurosciences, Universitat Autònoma de Barcelona, 01893 Bellaterra, Spain; nuria.gaja@uab.cat (N.G.-C.); neus.solanes@uab.cat (N.H.); 2 Centro de Investigación Biomédica en Red Sobre Enfermedades Neurodegenerativas (CIBERNED), 28031 Madrid, Spain; 3 Welab Barcelona, Parc Científic Barcelona, 08028 Barcelona, Spain; syeste@welab.barcelona (S.Y.); rreinoso@welab.barcelona (R.F.R.); jburgueno@welab.barcelona (J.B.); amontero@welab.barcelona (A.M.); mmerlos@welab.barcelona (M.M.); jvela@welab.barcelona (J.M.V.)

**Keywords:** amyotrophic lateral sclerosis, motoneuron degeneration, sigma-1 receptor, SOD1^G93A^ mice, spinal nerve injury

## Abstract

Motor neuron diseases (MNDs) include sporadic and hereditary neurological disorders characterized by progressive degeneration of motor neurons (MNs). Sigma-1 receptor (Sig-1R) is a protein enriched in MNs, and mutations on its gene lead to various types of MND. Previous studies have suggested that Sig-1R is a target to prevent MN degeneration. In this study, two novel synthesized Sig-1R ligands, coded EST79232 and EST79376, from the same chemical series, with the same scaffold and similar physicochemical properties but opposite functionality on Sig-1R, were evaluated as neuroprotective compounds to prevent MN degeneration. We used an in vitro model of spinal cord organotypic cultures under chronic excitotoxicity and two in vivo models, the spinal nerve injury and the superoxide dismutase 1 (SOD1)^G93A^ mice, to characterize the effects of these Sig-1R ligands on MN survival and modulation of glial reactivity. The antagonist EST79376 preserved MNs in vitro and after spinal nerve injury but was not able to improve MN death in SOD1^G93A^ mice. In contrast, the agonist EST79232 significantly increased MN survival in the three models of MN degeneration evaluated and had a mild beneficial effect on motor function in SOD1^G93A^ mice. In vivo, Sig-1R ligand EST79232 had a more potent effect on preventing MN degeneration than EST79376. These data further support the interest in Sig-1R as a therapeutic target for neurodegeneration.

## 1. Introduction

Motor neuron diseases (MNDs) represent a heterogeneous group of chronic sporadic and hereditary neurological disorders involving the upper and/or lower motor neurons (MNs), mainly represented by amyotrophic lateral sclerosis (ALS) in adults and spinal muscular atrophy (SMA) in children. There is no effective treatment available yet for most of these diseases; three treatments based on gene therapy are approved for SMA, and only two drugs are approved for ALS, riluzole and edaravone, which slightly prolong the lifespan of the patients [1,2]. This is in good part due to the multiple etiopathogenetic mechanisms, with a proposed complex interplay between excitotoxicity, neuroinflammation, oxidative stress, protein aggregation, mitochondrial dysfunction, and axonal transport defects, contributing to MN degeneration [3].

Sigma-1 receptor (Sig-1R) is a protein enriched in the endoplasmic reticulum of MNs. It acts as a chaperone modulating several essential cellular processes [4,5], although its direct downstream signaling is not completely known. Sig-1R is encoded by the *SIGMAR1* gene, and several mutations on its gene have been identified that lead to different types of MND, such as a severe, juvenile-onset form of ALS (ALS16) [6], development of frontotemporal dementia (FTD)-ALS [7], and some familial cases of distal hereditary motor neuropathies (dHMNs) [8,9]. Several Sig-1R ligands have been used in therapeutic assays in a number of neurodegenerative diseases, including Alzheimer’s, Parkinson’s, and Huntington’s disease [10]. In addition, the administration of Sig-1R ligands was shown to exert neuroprotection in various experimental models of MN degeneration, including in vitro excitotoxicity [11,12] and in vivo models of ALS [12,13,14,15], spinal root injury [16,17], and the wobbler mouse, a model of spontaneous MN degeneration [18]. Taken together, all these data illustrate the connection between Sig-1R and MN survival.

The aim of this study was to evaluate the therapeutic effect of novel synthesized selective Sig-1R ligands, coded as EST79232 and EST79376, on well-characterized in vitro and in vivo models of MN degeneration. These compounds, different from others reported in the literature, were also used as tool compounds to elucidate the impact of Sig-1R functionality, in terms of agonistic or antagonistic interaction, on compound efficacy. Thus, the compounds were first tested in vitro to estimate the affinity to the Sig-1R and to define the pharmacological profile of both new compounds. Then, the compounds were evaluated in spinal cord organotypic culture (SCOC) under chronic excitotoxicity to determine the neuroprotective profile of both compounds. Finally, the two compounds were moved to assays in a nongenetic model of MN death induced by spinal nerve injury (rhizotomy) and in the transgenic SOD1^G93A^ mouse model of ALS. 

## 2. Results

### 2.1. In Vitro Pharmacological Profile of EST79232 and EST79376

**Figure 1:**
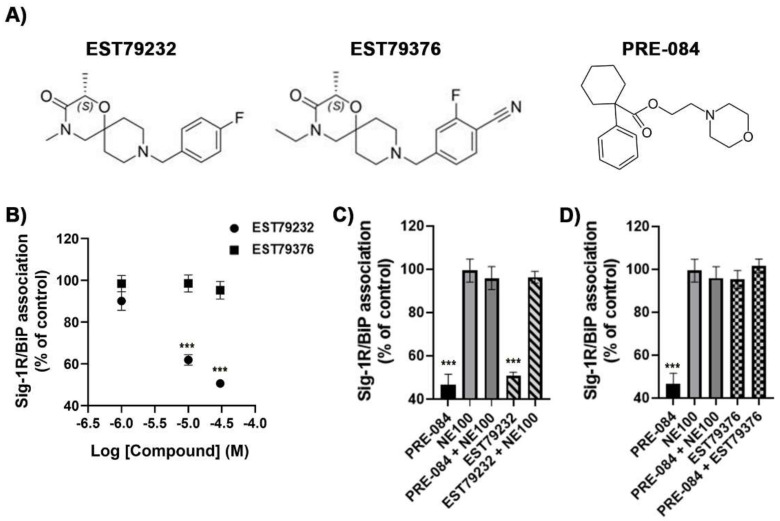
In vitro functionality of EST79232 and EST79376 at Sig-1R: Sig-1R and BiP interaction assay. (**A**) Chemical structure of the two new compounds synthesized, EST79232 and EST79376 from the same chemical series and PRE-084. (**B**) Concentration-response curves of EST79232 and EST79376 on the Sig-1R/BiP interaction. (**C**,**D**) Agonist effect of 10 µM of PRE-084, and the antagonist effect of 10 µM of NE100. NE100 was able to revert the effect of PRE-084. EST79232 at 30 μM was able to reduce the association between Sig-1R and BiP, and this effect was reverted by NE100 (**C**). EST79376 at 30 µM had no effect on its own, but it was able to revert the effect of PRE-084 (**D**). Values are expressed as mean ± SEM of 6 determinations. *** *p* < 0.001 vs. control. One-way ANOVA, followed by Dunnett’s post-hoc multiple comparisons test.

Radioligand assays revealed that the two new compounds synthesized, EST79232 and EST79376 (Figure 1A), were highly selective Sig-1R ligands. They showed high affinity for the human Sig-1R (Ki = 32 nM and Ki = 36 nM, respectively), while they did not show affinity for the human Sigma-2 receptor (Sig-2R) (IC50 > 10,000 nM for both). Similarly, PRE-084 has good affinity for the human Sig-1R (Ki = 72 nM) and no affinity for the human Sig-2R (IC50 > 10,000 nM). EST79232 and EST79376 also showed no significant affinity (% inhibition < 50% at 10 µM) for any of the targets included in the selectivity panel, apart from the Sig-1R.

Binding immunoglobulin protein (BiP)/Sig-1R association assays were used to determine the Sig-1R functional profile of EST79232 and EST79376, as described previously by Hayashi and Su [19]. EST79232 produced a concentration-dependent dissociation of Sig-1R from BiP, as observed with the prototypical Sig-1R agonist PRE-084, while EST79376 did not produce any change in the interaction between the two proteins (Figure 1B). Moreover, the agonistic effect of EST79232 on Sig-1R-BiP interaction could be reverted using the prototypical Sig-1R antagonist NE100 (Figure 1C), and the effect of PRE-084 could be reverted by EST79376 (Figure 1D). Based on BiP/Sig-1R association assay, EST79232 and EST79376 show agonist and antagonist functional profiles for Sig-1R, respectively.

### 2.2. EST79232 and EST79376 Prevent MN Death in SCOC under Chronic Excitotoxic Stress

**Figure 2:**
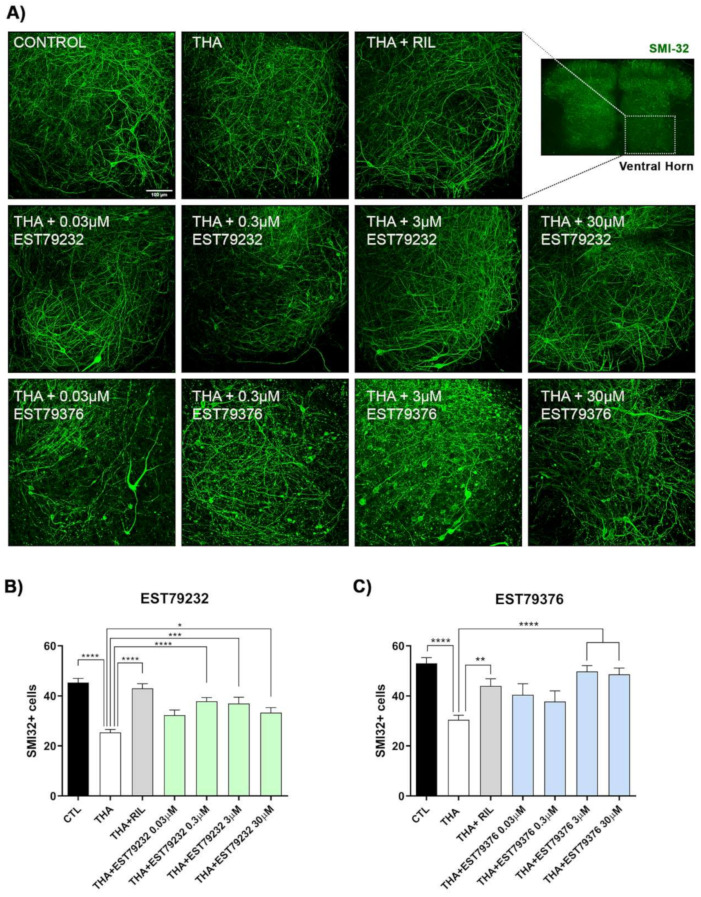
EST79232 and EST79376 ligands prevent MN death under chronic excitotoxicity. (**A**) Representative confocal images of spinal cord ventral horns labeled with SMI32 antibody at 28 DIV for control, THA and THA plus EST79232 or EST79376 treated at four concentrations (0.03, 0.3, 3, and 30 μM). Scale bar: 100 μm. (**B**,**C**) Bar graphs showing the number (mean ± SEM; *n* = 20–24 hemisections per treatment) of SMI-32-positive cells of each spinal cord hemislice. One-way ANOVA, followed by Bonferroni’s post-hoc test; **** *p* < 0.0001, *** *p* < 0.001; ** *p* < 0.01, * *p* < 0.05 vs. THA condition.

In the SCOC, the number of SMI32-positive MNs was significantly reduced in the ventral horn of slices treated with THA compared to the control slices (Figure 2). Riluzole, used as a positive control against excitotoxicity, preserved MNs at levels of control cultures. As shown in Figure 2B, slices treated with EST79232 had significantly more SMI-32-positive neurons at three concentrations tested, with the highest effect at 0.3 to 3 μM. The two highest concentrations of EST79376 assessed (3 and 30 μM) also significantly preserved MNs (Figure 2C).

### 2.3. EST79232 and EST79376 Enhance MN Survival after Rhizotomy

After rhizotomy surgery, nerve conduction tests were performed to confirm the complete and selective lesion of L4 and L5 spinal roots. There were no recordable compound muscle action potentials (CMAPs) from tibialis anterior (TA) and gastrocnemius (GM) muscles, innervated by axons of L4-L5 roots, ensuring complete denervation in all the mice included in the study. On the other hand, there was only partial preservation of the plantar interosseous muscle innervation, provided by L5-L6 roots, as in previous studies [17]. The corresponding contralateral muscles showed normal CMAPs. The mice presented an altered walking pattern, dragging the denervated right hindlimb during locomotion. The denervated muscles suffered severe atrophy. Functional outcomes did not change during the follow-up since no repair procedure was applied to the transected roots.

In the rhizotomy mice, plasma levels of EST79232 and EST79376 were detected after 15 min of the last i.p. administration (day 42 after surgery, end of the study) at 0.5 mg/kg and 5 mg/kg doses (Table 1).

**Figure 3:**
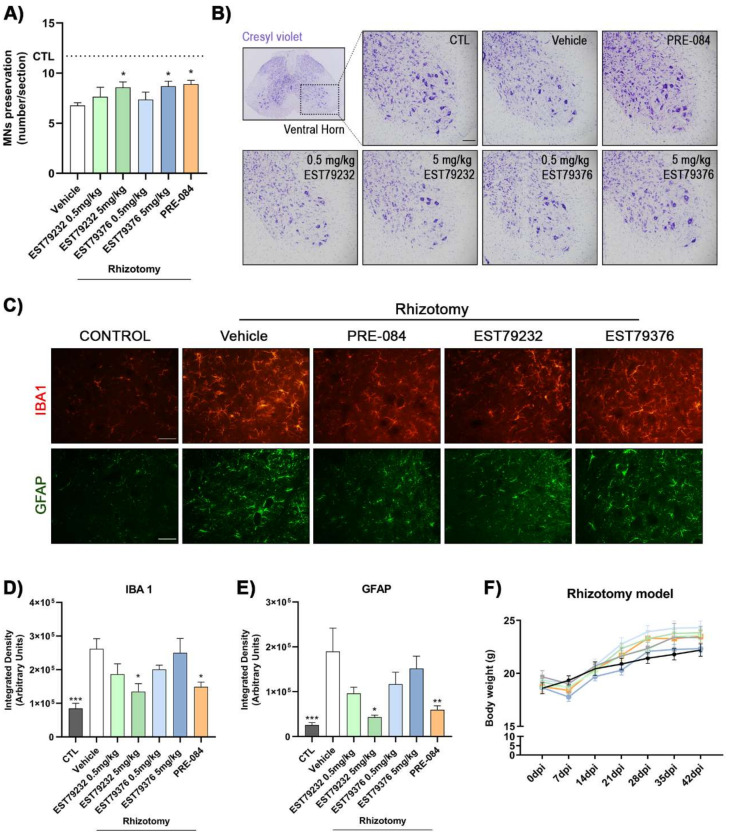
Administration of EST79232 and EST79376 increased MN survival and modulated glia activation after L4-L5 spinal roots injury. (**A**) Plot of number of α-MNs in L4-L5 segments, showing higher number of MNs in mice treated with Sig-1R ligands than in untreated ones. Animals per group: control (CTL), *n* = 14; vehicle, *n* = 14; PRE-084, *n* = 5. EST79232: 0.5 mg/kg, *n* = 5; 5 mg/kg, *n* = 6; EST79376: 0.5 mg/kg, *n* = 5; 5 mg/kg, *n* = 5. (**B**) Representative images corresponding to ventral horns of L4-L5 cord segments ipsilateral to rhizotomy in female mice with or without Sig-1R ligands treatment at 42 days post-injury (dpi). Scale bar: 100 μm. **C)** Representative images of glial reactivity assessed by microglia (IBA1) and astrocytes (GFAP) immunolabeling in the ventral horn of control and rhizotomized mice with vehicle or Sig-1R treatment (PRE-084, EST79232, and EST79376 at dose of 5 mg/kg) at 42 dpi. Scale bar: 50 μm. (**D**,**E**) Bar graph showing the integrated density of Iba-1 and GFAP immunolabeling in the ipsilateral ventral horn of spinal cord. *n* = 6 mice per group. (**F**) Plot of the body weight of rhizotomized mice during the study. All the mice groups presented a mild reduction in the body weight the first week after surgery, then gained weight normally during the follow-up. Data are mean ± SEM, analyzed with one-way (**A**,**D**,**E**) or two-way (**F**) ANOVA and Bonferroni’s multiple comparisons test. * *p* < 0.05, ** *p* < 0.001, *** *p* < 0.0001 vs. vehicle rhizotomized mice.

Histological analyses revealed that L4-L5 rhizotomy caused severe MN loss (43%) in the affected lumbar segments and that the three Sig-1R ligands reduced MN degeneration at 42 days after the injury. Rhizotomized mice treated during 6 weeks with EST79232 and EST79376 at a dose of 5 mg/kg/bid i.p. had a significantly higher number of spinal MNs (8.6 ± 0.6 and 8.7 ± 0.5, respectively; number of MNs ± SEM) compared to the untreated injured group (6.8 ± 0.3) in the ipsilateral ventral horn of spinal cord sections stained with cresyl violet (Figure 3A,B). The administration of these new Sig-1R ligands increased the number of surviving MNs at the same level as PRE-084 (8.9 ± 0.4), used as a positive control of MN preservation [14,16,17]. Administration of EST79232 and EST79376 at the low dose (0.5 mg/kg) did not exert significant MN protective effects (7.6 ± 0.9 and 7.4 ± 0.7, respectively) after rhizotomy.

As a consequence of the rhizotomy, microglia and astrocytes were activated surrounding lumbar MNs. Iba1 and GFAP immunostaining showed a marked increase in the untreated rhizotomized group (26 × 10^4^ ± 3 × 10^4^ and 19 × 10^4^ ± 5 × 10^4^, respectively; mean of integrated density ± SEM) compared with control mice (8 × 10^4^ ± 1.5 × 10^4^ and 2.5 × 10^4^ ± 0.6 × 10^4^) (Figure 3C–E). Regarding pharmacologic treatments, EST79232 at the highest dose significantly diminished microglial (13 × 10^4^ ± 2 × 10^4^) and astroglial (4 × 10^4^ ± 0.4 × 10^4^) reactivity at levels similar to the positive control PRE-084 (15 × 10^4^ ± 1.4 × 10^4^ and 6 × 10^4^ ± 0.9 × 10^4^). In contrast, EST79376 at 5 mg/kg did not modulate glial reactivity (25 × 10^4^ ± 4 × 10^4^ and 15 × 10^4^ ± 2.7 × 10^4^). The low doses tested of both Sig-1R ligands showed a nonsignificant tendency to decrease microgliosis and astrogliosis.

Finally, it is important to note that twice daily administration for 6 weeks of these new compounds did not result in noticeable side effects on mice, with neither signs of abnormal behavior nor difference in body weight compared with the vehicle group, thus suggesting that chronic administration of EST79232 and EST79376 did not cause overt toxicity in the animals (Figure 3F).

### 2.4. EST79232 Treatment Slows Disease Progression in SOD1^G93A^ Mice

The neuroprotective effect of the new Sig-1R ligands were also assessed in the SOD1^G93A^ mouse model of ALS. In this study, only the dose of 5 mg/kg of each compound was tested based on previous results in the rhizotomy model. After administration of 5 mg/kg i.p. for 8 weeks (from 8 to 16 weeks of age), plasma concentrations were determined 15 min after the last administration of both compounds, EST79232 and EST79376 (Table 1).

**Figure 4:**
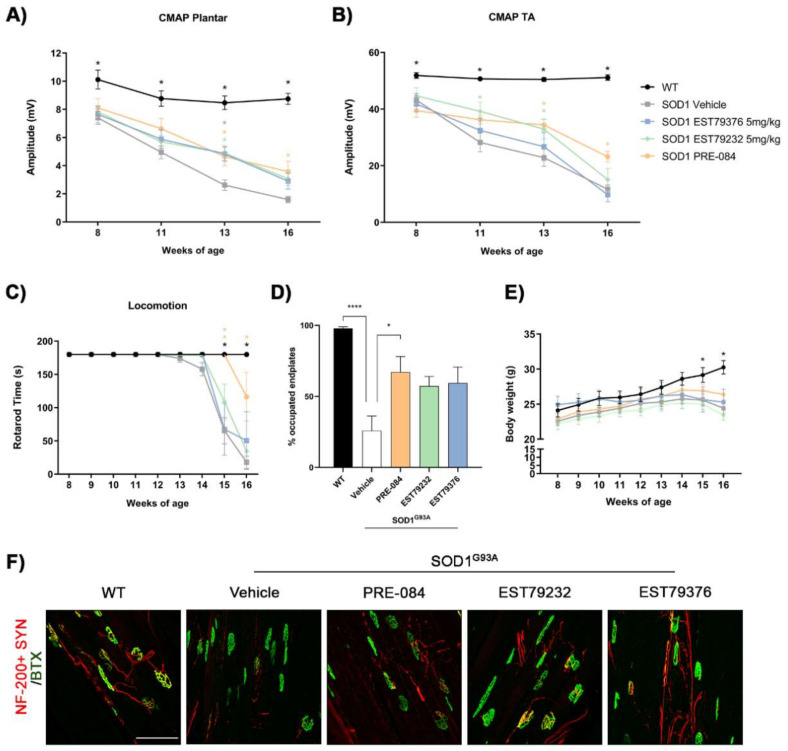
Effects of administration of EST79232 and EST79376 on disease progression of SOD1^G93A^ mice. (**A**,**B**) Plots of the CMAP amplitude of plantar and tibialis anterior (TA) muscles. I EST79232 treatment produced a slight improvement in the rotarod performance in treated SOD1^G93A^ mice (*n* = 9 SOD1 vehicle, *n* = 7 SOD1 EST79232, *n* = 5 SOD1 EST79376, and *n* = 5 SOD1 PRE-084 mice). (**D**) Histological analyses of NMJ showed a tendency to maintain the proportion of occupied endplates in the GM muscle of treated SOD1^G93A^ with the two novel Sig-1R ligands compared to vehicle group (*n* = 5 per group). (**E**) Plot of the body weight of the different groups of mice during the follow-up. At 15 and 16 weeks, there was a significant difference between SOD1^G93A^ mice and wild-type (WT) mice. (**F**) Representative confocal images of NMJ in the GM muscle of WT mice and SOD1^G93A^ male mice with or without Sig-1R ligands treatment at 16 weeks of age. Scale bar: 100 μm. Data are mean ± SEM, analyzed with one-way (**D**) or two-way (**A**–**C**,**E**) ANOVA with Bonferroni’s multiple comparisons test. **** *p* < 0.0001, * *p* < 0.05 vs. SOD1^G93A^ vehicle.

Electrophysiological tests were performed from 8 to 16 weeks of age to assess MN function. At the early (11 weeks) and mid (13 weeks) stages of the disease, motor nerve conduction tests showed that administration of EST79232 significantly prevented the decline in amplitude of the CMAP of TA and plantar muscles, similar to PRE-084. However, at 16 weeks, the effect was reduced to levels found in the vehicle group (Figure 4A,B). Mice treated with EST79376 only showed significant preservation of the CMAP amplitude in the plantar muscle at 13 weeks of age compared to the vehicle group. Regarding the latency of CMAPs, the values in all the SOD1^G93A^ mice groups were slightly longer than in wild-type (WT) mice during the follow-up, but there were no significant differences between the disease groups (Supplementary Materials Figure S1).

Results of the rotarod test showed that animals treated with both new Sig-1R ligands had a delay of one week (15 weeks) in the onset of motor impairment in comparison with untreated animals (14 weeks). When compared, EST79232 produced a significant improvement of SOD1G93A mice in the rotarod performance at 15 weeks of age, but PRE-084 treatment significantly improved the functional outcome at 15 and 16 weeks (Figure 4C).

The histological analyses of neuromuscular junctions (NMJs) of the hindlimb muscle showed that PRE-084 treatment significantly increased the proportion of innervated endplates compared to untreated SOD1^G93A^ mice at 16 weeks, consistent with the electrophysiological results of higher CMAP amplitude values. EST79232 and EST79376 caused a tendency to preserve NMJ innervation, although differences did not reach statistical significance when compared with the vehicle group (*p* = 0.081 and *p* = 0.057, respectively) (Figure 4D–F).

Similar to the findings in the rhizotomy study, daily i.p. administration of the Sig-1R ligands EST79232, EST79376, and PRE-084 to SOD1^G93A^ mice did not cause differences in body weight compared with the vehicle group (Figure 4E).

### 2.5. EST79232 Treatment Protects Spinal MNs and Reduces Astroglial Activation in SOD1^G93A^ Mice

**Figure 5:**
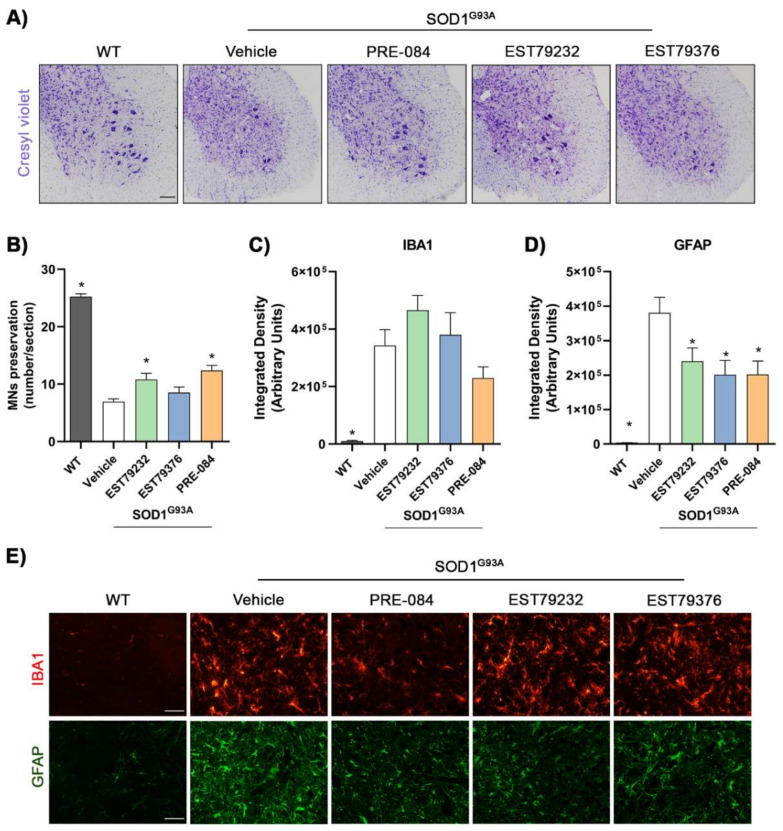
Neuroprotective effects of Sig-1R ligands administration on MNs and glial reactivity in SOD1^G93A^ mice at 16 weeks. (**A**) Representative images of the ventral horns stained with cresyl violet of WT and SOD1^G93A^ male mice with or without Sig-1R ligands treatment. Scale bar: 100 μm. (**B**) Histological analyses showed an increase in number of MNs in mice treated with EST79232 and PRE-084. Animals per group: WT, *n* = 13; SOD vehicle, *n* = 16; SOD PRE-084, *n* = 5; SOD EST79232, *n* = 7; SOD EST79376, *n* = 5. (**C**,**D**) Bar graph showing the integrated density of Iba-1 and GFAP immunolabeling in the ventral horn of spinal cord. *n* = 5–10 mice per group. Data are expressed as mean ± SEM and analyzed by one-way ANOVA and Bonferroni’s multiple comparisons test. * *p* < 0.05 vs. SOD1^G93A^ vehicle mice. (**D**) Representative images of glial reactivity assessed by IBA and GFAP to label microglia and astrocytes in the ventral horn. Scale bar: 50 μm.

Counts of α-MNs in ventral horns of spinal cord sections revealed that untreated SOD1^G93A^ mice had a loss of around 60% MNs (6.9 ± 0.5 MNs per section) compared to WT mice (25.3 ± 0.5) at 16 weeks of age. SOD1^G93A^ mice treated with EST79232 (5 mg/kg) had a significantly higher number of surviving spinal MNs (10.8 ± 1.1), as the animals were administered with PRE-084 (12.4 ± 0.9), whereas EST79376 did not produce a significant effect (8.5 ± 1.0) (Figure 5A,B). Glial reactivity showed a marked increase in SOD1^G93A^ compared to WT mice. Treatment with the new Sig-1R ligands EST79232 and EST79376 produced a significant decrease in astrocyte activation as did PRE-084, whereas they did not reduce microglial activation (Figure 5C–E).

## 3. Discussion

The results of this study show that two novel Sig-1R ligands, coded as EST79232 and EST79376, exert neuroprotective effects in three different experimental models of MN degeneration, hence adding to previous studies highlighting Sig-1R as a promising target to treat MND. These two new synthesized compounds have high affinity for the human Sig-1R and high selectivity, as proved by the lack of affinity against a panel of other 180 targets, including Sig-2R. These compounds may be advantageous to avoid interfering and/or adverse effects due to the binding to other receptors (off-target effects). Therefore, neuroprotective activity found in this study is attributable to Sig-1R binding.

Sig-1R is implicated in multiple cellular processes, such as calcium release modulation through inositol triphosphate receptor type 3 (IP_3_R) stabilization, endoplasmic stress regulation with the formation of a complex with BiP protein, and cell survival [20,21]. Several studies have supported a neuroprotective role of Sig-1R activation by administering an agonist ligand, on neurodegenerative disorders, including Alzheimer’s disease, Parkinson’s disease, Huntington’s disease, ALS, stroke, retinal degeneration, and depression [13,14,22,23,24,25]. On the other hand, blocking Sig-1R by administration of antagonists was shown useful for the amelioration of psychosis, pain, drug abuse, and cancer [26].

In the present study, we used the in vitro model of SCOC to assess the potential to protect against chronic excitotoxicity as a drug screening before testing promising compounds on in vivo models [27,28]. Both novel Sig-1R compounds tested exerted neuroprotective effects by reducing MN death, at more than one concentration (EST79232 at 0.3 to 30 μM and EST79376 at 3 to 30 μM). Such a bell-shaped dose-dependent protective effect on MN death induced by excitotoxicity in SCOCs was also previously reported for the agonist PRE-084 [11]. SA4503, another Sig-1R agonist, reduced NSC34 SOD1^G93A^-induced cell death in a concentration-dependent manner, with a more significant effect at 10 μM [12]. On the contrary, the Sig-1R antagonists BD1063 and BD1047 abolished the protective effect obtained with PRE-084 and SA4503, respectively [11,12]. In our study, neuroprotective effects were observed with the compounds EST79232 and EST79376, classified as Sig-1R agonist and antagonist, respectively, based on BiP binding assay. Recently, it has been described that the Sig-1R ligands SA4503, an agonist, and BD1063, an antagonist, exert neuroprotection in SCOCs under chronic excitotoxicity [15]. These results highlight the importance of Sig-1R modulation by ligands independently on their classification as agonists or antagonists, especially because ligand functionality classification may depend on the assay and, importantly, on the dose (bi-phasic/bell-shaped dose–response effect, with an antagonism profile at higher doses being a common feature in numerous preclinical models in vitro, in vivo, and in clinical trials) [29]. Furthermore, Tadić et al. (2017) demonstrated that Sig-1R agonists, SA4503 and PRE-084, act differently enhancing the cytosolic calcium exchange in SOD1^G93A^ mice cultured MNs [30]. Thus, there is no clear consensus on the type of Sig-1R ligands that may be more effective to prevent neurodegeneration, highlighting the need to evaluate their effects in reliable models.

A proximal nerve injury, at the root or spinal nerve level, results in the death of a significant number of MNs [16,17,31], as in the L4-L5 rhizotomy model. We reported that the Sig-1R agonists PRE-084 and SA4503 and the antagonist BD1063 enhanced MN preservation in this model through IRE1-XBP1 activation and modulation of glia reactivity [17]. Similarly, administration of EST79376 and EST79232 at the dose of 5 mg/kg produced an increase in the number of surviving lumbar spinal MNs, similar to PRE-084, indicating that modulating the Sig-1R represents a good strategy to prevent MN death and extend the time window for surgical repair after spinal root and plexus injuries.

In the SOD1^G93A^ transgenic mice, the most commonly used ALS model, we found that daily administration of EST79232 from 8 to 16 weeks of age improved functional outcomes and delayed the onset of disease. EST79232 treatment also increased the number of surviving MNs and showed a tendency to preserve innervated NMJ in treated SOD1^G93A^ mice. The results of EST79232 treatment were similar to those obtained with the prototypic agonist PRE-084. Accordingly, PRE-084 is known to exert an enhancing effect on the preservation of spinal MNs and extend the lifespan in the SOD1^G93A^ mouse [14]. Beneficial effects on SOD1^G93A^ mice were also observed with the Sig-1R agonist SA4503, which extended the survival time compared with untreated animals [12]. Additionally, the antagonist BD1063 was able to preserve the neuromuscular function of the hindlimbs and increased the number of surviving MNs in treated female SOD1^G93A^ mice [15]. Interestingly, we found that treatment with EST79376 also produced positive effects, although less marked than with EST79232 and PRE-084, despite its putative consideration as a Sig-1R antagonist. Curiously, the best results overall were obtained with the ligand PRE-084, which has less affinity for the Sig-1R than the two newly developed compounds. This comparison points to the complexity of the Sig-1R modulation. Further studies are needed to better understand the function of this receptor and the particularities driving the actual action of its ligands.

In addition to MNs, microglia and astrocytes also express Sig-1R [32]. Glial cell reactivity is associated and has been proposed to play a causative role in MN death following root lesions [31] and also in MND models [33,34,35]. Thus, reducing glial activation may help to ameliorate the deleterious spinal MN environment in these conditions. Our results show that treatment with EST79232 and with PRE-084 reduced glial reactivity after rhizotomy. Similarly, other Sig-1R ligands, including PRE-084, BD1063 and SA4503, reduced astrogliosis after spinal root injuries [16,17]. PRE-084 treatment was reported to reduce microglia activation in SOD1^G93A^ mice [14]. In contrast, we found that EST79232 and EST79376 had an effect on reducing astroglia activation in the ALS model but no effect on microglial cells. In the spinal muscular atrophy Smn^2B/-^ mice, PRE-084 attenuated reactive astrogliosis and restored the imbalance of M1/M2 microglia, although no improvement in clinical outcome was observed [36]. Therefore, treatment with Sig-1R ligands may contribute to regulating the deleterious role of activated glial cells in MN degenerative conditions, although the precise outcome of phenotypic modulation merits further investigation.

## 4. Materials and Methods

### 4.1. Human Sig-1R Radioligand Assay

The binding properties of the compounds to human Sig-1R were studied in transfected HEK-293 membranes using [^3^H](+)-pentazocine (NET-1056,PerkinElmer; Waltham, MA, USA) as the radioligand. The assay was carried out with 7 μg of membrane suspension, [^3^H](+)-pentazocine (5 nM, 100 μL), in either the absence or presence of either buffer or 10 μM haloperidol for total (TB) and nonspecific binding (NSB), respectively, or the corresponding compound concentration. Binding buffer contained Tris-HCl (50 mM, pH 8). Plates were incubated at 37 °C for 120 min. After the incubation period, the reaction mix was transferred to MultiScreen HTS, FC plates (Merck Millipore; Carrigtohill, Ireland) presoaked in 0.1% polyethyleneimine and filtered. Then, plates were washed with ice-cold Tris-HCl (10 mM, pH 7.4). Filters were dried and counted at approximately 40% efficiency in a MicroBeta scintillation counter (PerkinElmer) using an EcoScint liquid scintillation cocktail. The MicroBeta reader gave the counts per minute (cpm) per each well. The data were entered in ActivityBase, and a convenient TestSet (XLfit) allowed us to obtain means of duplicates, calculation of Specific Binding obtained by subtracting NSB from TB, % of inhibition for each tested compound, values for the IC50 (nM) and the Ki (nM), and a graph representation. Percentages of binding (specific, nonspecific, and total) for each different compound concentration were calculated ((Compound total binding—NSB)/(TB-NSB)) × 100). Inhibition constant (Ki) was calculated from IC50 according to the Cheng–Prusoff equation (Ki = IC50/(1 + L/Kd), in which L is the radioligand concentration, determined from the experimental total counts using the specific activity of the radioligand, and Kd is the dissociation constant of the radioligand [37].

### 4.2. Human Sig-2R Radioligand Assay

The binding properties of the compounds to the human Sig-2R were similarly studied in transfected HEK-293 Sig-1R knockout membranes using [^3^H]-1,3-di-o-tolylguanidine (DTG) (NET-986, PerkinElmer; Boston, USA) as the radioligand. The assay was carried out with 15 μg of membrane suspension, [^3^H]-1,3-di-o-tolylguanidine (DTG) (10 nM, 100 μL), in either the absence or presence of either buffer or 10 μM haloperidol for total (TB) and nonspecific (NSB) binding, respectively, or the corresponding compound concentration. Binding buffer contained Tris-HCl (50 mM, pH 7.4). Plates were incubated at 25 °C for 120 min. After incubation, the reaction mix was transferred to MultiScreen HTS, FC plates (Merck Millipore) presoaked in 0.5% polyethyleneimine, and filtered. Following that, plates were washed with ice-cold Tris-HCl (10 mM, pH 8). Filters were dried and counted, and data were processed as above [37].

### 4.3. Selectivity Profile

Binding affinities of EST79232 and EST79376 for proteins other than Sig-1R and Sig-2R were determined by commercial radioligand binding assays by EurofinsPanlabs. A selectivity profile including a panel of more than 180 radioligand binding assays for different receptors, ion channels, enzymes, and transporters was performed according to their standard in vitro screening assay protocols (http://www.eurofinspanlabs.com (accessed on 15 May 2022)). Assays were performed with concentrations tested in duplicate.

### 4.4. BiP/Sig-1R Association Assay

The interaction between Sig-1R and the chaperone BiP was used to identify the functional nature (agonistic or antagonistic) of compounds [38]. The assay was conducted by Amylgen according to their standard assay protocol (https://www.amylgen.fr/ (accessed on 15 May 2022)). Each assay condition consisted of six determinations. In brief, CHO cells were treated with compounds at 37 °C for 30 min and lysed. CHO cell lysates were immunoprecipitated with Sig-1R antibody, and coimmunoprecipitated BiP was detected by ELISA assay. In each experiment, untreated control cell lysates were used to assess the maximal level of Sig1R-BiP interaction and represented 100% of Sig1R-BiP association. Cells treated with compounds at the corresponding concentrations were equally processed, and the level of Sig1R-BiP association in those conditions was referred to as the control condition. In this experimental setting, Sig1R agonists were able to reduce the Sig1R-BiP interaction, while antagonists were not.

### 4.5. Spinal Cord Organotypic Cultures (SCOCs)

SCOCs from lumbar sections of 8-day-old Sprague–Dawley rats were prepared as previously described [39]. Briefly, the spinal cord was collected and cut into 350 μm thick transverse sections with a McIlwain Tissue Chopper. Four lumbar sections were transferred onto Millicell-CM nets (0.4 μm, PICM03050, Millipore) in medium (50% (*v*/*v*) minimal essential medium (MEM, M5775, Sigma), 2 mM glutamine, 25 mM HEPES, 25% (*v*/*v*) Hank’s Balanced Salt Solution (HBSS^−/−^;14175, Gibco; UK) supplemented with 25.6 mg/mL glucose, and 25% (*v*/*v*) heat-inactivated horse serum (26050-088, Gibco; New Zealand), pH = 7.2). Cultures were maintained at 37 °C in a 5% CO_2_ humidified cabin and left to stabilize. After 7 days in vitro (DIV), DL-threo-β-hydroxyaspartic acid (THA; 100 μM: H2775, Sigma Aldrich, St. Louis, MI, USA) was added to induce chronic excitotoxicity [28]. The neuroprotective effect of each Sig-1R ligand was assessed by its coaddition to the culture with THA and renewing at each medium change. EST79232 (in DMSO 100 mM) and EST79376 (in PBS 1 mM) were tested at four concentrations (30, 3, 0.3, and 0.03 μM). Comparisons were performed against slices with vehicle as negative control and with the addition of riluzole (5 μM) as positive control [11,27]. In vitro experiments were performed in three independent cultures, with at least 12 different SCOCs for each experimental condition.

Slices were maintained for 28 DIV and then fixed with 4% PFA. Fixed SCOC slices were immunolabeled with primary antibody mouse anti-neurofilament H nonphosphorylated (SMI-32; 1:500; 801701, BioLegend, San Diego, CA, USA), washed, and incubated with secondary antibody Alexa Fluor 488 donkey anti-mouse (1:500; A-21202, Invitrogen; Eugene, OR, USA) [15]. Cell nuclei were labeled with DAPI (1:2000) and the sections mounted with Fluoromount-G medium (SouthernBiotech, Birmingham, AL, USA). Images of the ventral horn were captured with a confocal microscope (LSM 700 Axio Observer, Carl Zeiss 20x/z0.5). MN survival was assessed by counting all SMI-32-positive neurons in each spinal cord using the Cell Counter plugin of ImageJ software.

### 4.6. Animals

For spinal nerve injury studies, adult female mice with B6SJL background were used, whereas for the ALS murine model, transgenic male mice carrying the G93A human mutation in the SOD1 gene (B6SJL-Tg[SOD1-G93A]1Gur) and nontransgenic wild-type (WT) littermates as controls were used [40]. The transgenic offspring was identified by polymerase chain reaction (PCR) amplification of DNA extracted from the tail. Mice were kept under standard conditions and handled in accordance with the guidelines of the European Union Council (Directive 2010/63/EU) and Spanish regulations on the use of laboratory animals. All experimental procedures were approved by the Ethics Committee of the Universitat Autònoma de Barcelona (CEEAH-UAB: 2969 and 4273).

### 4.7. Rhizotomy Procedure

Surgeries were performed on three-month-old mice under anesthesia with ketamine-xylazine (100–10 mg/kg i.p.) as previously described [17]. Briefly, the L4-L5 spinal roots were exposed by a small laminectomy on the right side. Afterwards, roots were cut at the exit from the intervertebral foramina, and the root stumps were separated. Mice were cared for until recovery in a warm environment. Analgesia was provided with buprenorphine (0.1 mg/kg) for the next 48 h post-surgery.

### 4.8. Drug Administration

**Figure 6:**
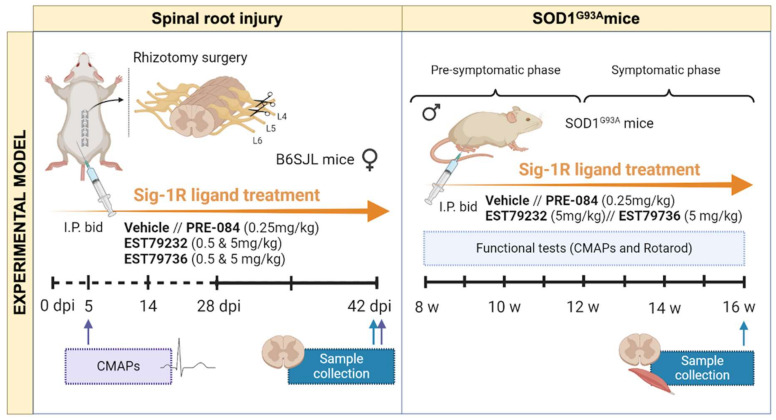
Schema of the in vivo studies. In the spinal root injury, treatment was given from the surgery until 42 days, when samples of the spinal cord were obtained. In the SOD1^G93A^ mice, EST79232 and EST79376 were given from 8 to 16 weeks of age. Electrophysiological and rotarod tests were performed periodically until 16 weeks, when samples of spinal cord and muscle were harvested.

The following selective Sig-1R ligands were used: PRE-084 at a dose of 0.25 mg/kg (0589,TOCRIS; Bristol, UK), EST79232 and EST79376 at doses of 0.5 and 5 mg/kg (synthesized and supplied by ESTEVE Pharmaceuticals). The compounds were dissolved in 0.5% hydroxypropyl-methylcellulose (HPMC; H7509, Sigma-Aldrich) in distilled water and were administered by intraperitoneal (i.p.) route twice daily (bid) in a volume of 10 mL/kg. Administrations were given from 30 min after rhizotomy surgery until 42 days post-injury (dpi), the end of the study; in the SOD1^G93A^ study, treatments were given from 8 to 16 weeks of age (see Figure 6).

For the spinal nerve injury (rhizotomy, rhizo), female WT mice were distributed in the following experimental groups: uninjured control (CTL) (*n* = 16), rhizo + vehicle 0.5% HPMC (*n* = 14), rhizo + PRE-084 0.25 mg/kg (*n* = 5), rhizo + EST79232 0.5 mg/kg (*n* = 5), rhizo + EST79232 5 mg/kg (*n* = 6), rhizo + EST79376 0.5 mg/kg (*n* = 5), rhizo + EST79376 5 mg/kg (*n* = 5). For the SOD1^G93A^ studies, male transgenic mice were divided into 4 groups: SOD1+ vehicle 0.5% HPMC (*n* = 13), SOD1 + PRE-084 0.25 mg/kg (*n* = 5), SOD1 + EST79232 5 mg/kg (*n* = 7), SOD1+ EST79376 5 mg/kg (*n* = 6), with B6SJL male WT age-matched mice used as negative control of the disease (*n* = 13).

### 4.9. Plasma Levels Associated with Pharmacological Activity

Plasma levels were determined in the two in vivo models evaluated to be able to associate the pharmacological activity with the levels present in plasma. In the rhizotomy model, the plasma levels were evaluated at Day 42 after surgery and at 15 min after compound administration (0.5 and 5 mg/kg i.p.). In the SOD1^G93A^ model, the plasma levels were evaluated at 16 weeks of age (after 8 weeks of administration) and 15 min after compound administration (5 mg/kg i.p.). Plasma samples were assayed by ultraperformance liquid chromatography−triple quadrupole mass spectrometry (UPLC-MS/MS) after plasma protein precipitation. Results are expressed as mean ± standard deviation (*n* = 8).

### 4.10. Electrophysiological Tests

Motor nerve conduction tests were performed before the surgery to obtain baseline values and at the end of the follow-up in the rhizotomy model, whereas the SOD1^G93A^ mice were evaluated at 8 weeks (prior to starting drug administration) and then every 2–3 weeks until the end point at 16 weeks of age (Figure 6). The sciatic nerve was stimulated by means of single pulses of 50 µs duration delivered from a Grass S88 stimulator through needle electrodes placed at the sciatic notch. The CMAP was recorded from TA, GM, and plantar interossei muscles with microneedle electrodes, with the active electrode being placed in the muscle and the reference at the fourth toe [41]. The CMAPs were amplified and displayed on a digital oscilloscope (Tektronix TD420S, Tektronix, Wilsonville, OR, USA) at appropriate settings to measure the latency and the amplitude from baseline to the maximal negative peak. The mice were anesthetized with pentobarbital (50 mg/kg i.p.), and their body temperature was maintained by means of a thermostatic warming pad.

### 4.11. Locomotion Tests

A rotarod test was performed to evaluate motor coordination and strength. The time that each animal remained on the rotating rod at a speed of 14 rpm was measured for five trials per mouse, and the longest time until falling was recorded; 180 s was chosen as the cut-off time [42]. The test was performed weekly from 8 to 16 weeks of age in SOD1^G93A^ and WT mice, and disease onset was determined as the first week when each mouse was unable to keep walking for 180 s on the rod.

### 4.12. Histological and Immunofluorescence Analyses

At the end of follow-up (42 dpi for rhizotomy and 16 weeks of age for ALS mice; Figure 6), the animals were deeply anesthetized and transcardially perfused with 4% PFA in PBS. The lumbar spinal cord and the TA muscle were harvested and cryopreserved in 30% sucrose solution in PB. The spinal cords were serially cut in 20 µm thick transverse sections with a cryostat (Leica, Mannheim, Germany). For MN counting, L4-L5 spinal cord sections separated 100 µm were stained for 3 h with an acidified solution of 3.1 mM cresyl violet. MNs were identified by their localization in the lateral ventral horn of the spinal cord and were counted following strict size and morphological criteria: only MNs with a diameter larger than 20 µm, polygonal shape, and prominent nucleoli were counted [14,17].

For glial cells immunofluorescence, other lumbar spinal cord sections were blocked with 10% normal donkey serum and incubated overnight with primary antibodies rabbit anti-Iba1 (1:500; 019–19741, Wako) and rat anti-GFAP (1:500; 13-0300, Invitrogen) to label microglia and astroglia, respectively. After several washes, sections were incubated with secondary antibodies Alexa Fluor 488-donkey anti-rat (1:500; A-21208, Invitrogen) or Alexa Fluor 594 donkey anti-rabbit (1:500; A21207, Invitrogen). Finally, sides were mounted with Fluoromount-G. To quantify glial cell reactivity, images of the ventral horn were acquired with an epifluorescence microscope (Nikon Eclipse Ni, Japan) using the same conditions for each analyzed marker. After defining a threshold for background correction, the integrated density of GFAP or Iba1 labeling was measured using ImageJ software.

For neuromuscular junction (NMJ) labeling, 50 µm thick longitudinal sections of TA muscle were serially cut. Sections were blocked with 5% normal donkey serum and incubated with primary antibodies rabbit anti-synaptophysin (1:500; Ab32127, Abcam, Cambridge, UK) and chicken anti-neurofilament 200 (NF200, 1:1000; AB5539, Millipore, Burlington, MS, USA) 48 h at 4 °C. After washes, sections were incubated overnight with secondary antibody Alexa Fluor 594-donkey anti-rabbit and anti-chicken (1:200; A11042-A21207, Invitrogen, USA) and Alexa 488 conjugated α-bungarotoxin (1:500; B13422, Life Technologies, Carlsbad, CA USA). Confocal images were captured (LSM 700 Axio Observer, Carl Zeiss, Jena, Germany, 40xOil/z0.5), and the maximum projection images generated from 1.3 µm z projections. The proportion of innervated NMJs was calculated by classifying each endplate as occupied (when presynaptic terminals overlie the endplate) or vacant (no presynaptic label in contact with the endplate). At least 60 endplates were analyzed per muscle.

### 4.13. Statistical Analysis

All data are expressed as mean ± standard error of the mean (SEM). Statistics were performed using GraphPad Prism 6 software. Histological data were analyzed using one-way ANOVA followed by Bonferroni’s post-hoc test for multiple comparisons, and for in vitro pharmacological profile, Dunnett’s post-hoc multiple comparisons was used. Electrophysiological and functional measurements were statistically analyzed using one-way or two-way ANOVA, followed by Bonferroni’s post-hoc test for multiple comparisons. Statistical significance was set at *p* < 0.05.

## 5. Conclusions

The neuroprotective effect on MN degeneration of two new potent and selective Sig-1R ligands is reported. In vitro treatment with the agonist EST79232 and antagonist EST79376 significantly reduced MN death caused by chronic excitotoxicity. In vivo, Sig-1R ligand EST79232 had a more potent effect on preventing MN degeneration than EST79376. In addition, administration of EST79232 had effects on reducing astroglial reactivity in both models, and EST79376 only in the SOD1^G93A^ mouse model. The observation that Sig-1R ligands exert protective/preventive effects on different experimental models of MN degeneration opens promising perspectives for targeting Sig-1R for MND.

## Figures and Tables

**Table 1:** Plasma levels of EST79232, EST79376, and PRE-084 after chronic intraperitoneal administration.

Model	Compound	Dose (mg/kg)	Plasma Concentration (ng/mL)15 min Post-Administration	
			Mean	SD
**Spinal Nerve Injury female mouse**	**EST79232**	0.5	20	8
		5	265	53
	**EST79376**	0.5	21	7
		5	592	176
	**PRE-084 ^a^**	0.25	3.6	0.8
**Transgenic SOD1^G93A^ male mouse**	**EST79232**	5	425	129
	**EST79376**	5	692	206
	**PRE-084 ^a^**	0.25	2.6	1.3

^a^: Concentrations determined at 30 min post-administration. Data obtained in a separate study.

## Data Availability

The data that support the findings of this study are available from the corresponding author upon reasonable request.

## Supplementary Materials

The following are available online at https://www.mdpi.com/article/10.3390/ijms23126737/s1.
