# Supplementary material for: EST79232 and EST79376, Two Novel Sigma-1 Receptor Ligands, Exert Neuroprotection on Models of Motoneuron Degeneration
Source: Int J Mol Sci. 2022 Jun 16;23(12):6737. doi: 10.3390/ijms23126737 (PMC9223397; doi:10.3390/ijms23126737)
Supplement: Supplementary file 1 [file ijms-23-06737-s001.zip › ijms-1751272-supplementary.pdf]

International Journal of *Molecular Sciences*

MDPI

EST79232 and EST79376, Two Novel Sigma-1 Receptor Ligands, Exert Neuroprotection on Models of Motoneuron Degeneration

Núria Gaja-Capdevila [1,2], Neus Hernández [1,2], Sandra Yeste [3], Raquel F. Reinoso [3], Javier Burgueño [3], Ana Montero [3], Manuel Merlos [3], José M. Vela [3], Mireia Herrando-Grabulosa [1,2,*] and Xavier Navarro [1,2,*]

SUPPLEMENTARY FIGURE:

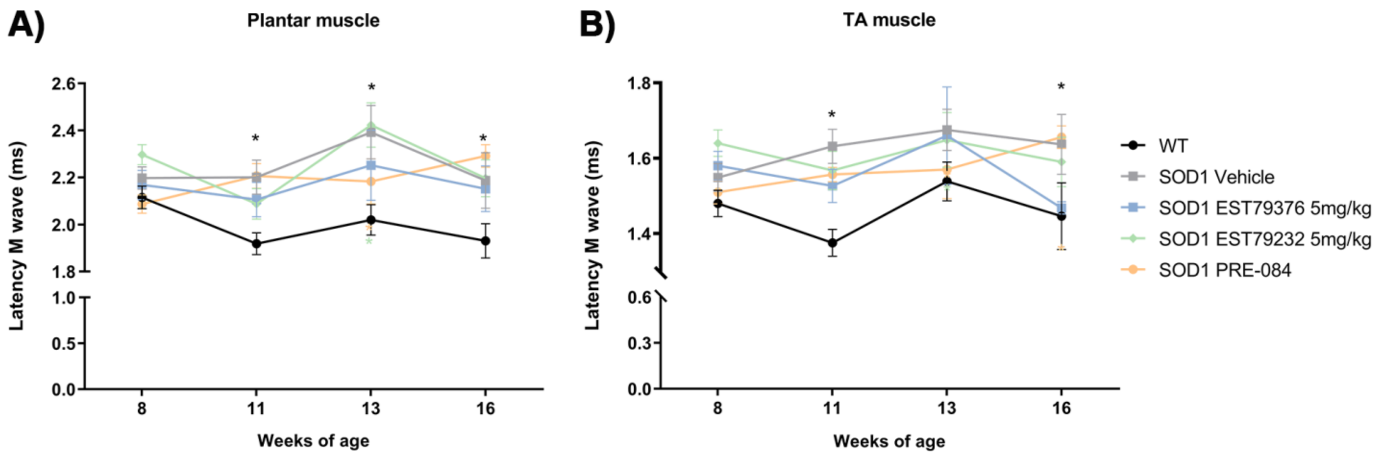

Figure S1. Latency results of nerve conduction tests. Plots of the latency to the peak of the CMAP of plantar interosseous (**A**) and tibialis anterior (TA) (**B**) muscles in wild type and in treated SOD1^{G93A} mice (n=9 SOD1 vehicle, n=7 SOD1 EST79232, n=5 SOD1 EST79376 and **n**=5 SOD1 PRE-084 mice). Data are mean ± SEM, analyzed with two-way ANOVA with Bonferroni's multiple comparisons test. *$p < 0.05$ versus WT.
